# Evaluation of The Combined Effects of Hyperthermia, Cobalt-60 Gamma Rays and IUdR on Cultured Glioblastoma Spheroid Cells and Dosimetry Using TLD-100

**Published:** 2014-10-04

**Authors:** Ali Neshasteh-Riz, Rozhin Rahdani, Ahmad Mostaar

**Affiliations:** 1 Department of Radiobiology, Iran University of Allied Health, Tehran, Iran; 2 Department of Nuclear Engineering, Science and Research Islamic Azad University, Tehran, Iran; 3 Department of Medical Physics, Shahid Beheshti University of Medical Sciences, Tehran, Iran

**Keywords:** Radiation, Hyperthermia, IUdR, Glioblastoma, Comet Assay

## Abstract

**Objective:**

In radiation treatment, the irradiation which is effective enough to control the tumors far exceeds normal-tissues tolerance. Thus to avoid such unfavourable outcomes, some methods sensitizing the tumor cells to radiation are used. Iododeoxyuridine (IUdR) is a halogenated thymidine analogue that known to be effective as a radiosensitizer in human cancer therapy. Improving the potential efficacy of radiation therapy after combining to hyperthermia depends on the magnitude of the differential sensitization of the hyperthermic effects or on the differential cytotoxicity of the radiation effects on the tumor cells. In this study, we evaluated the combined effects of IUdR, hyperthermia and gamma rays of ^60^Co on human glioblastoma spheroids culture.

**Materials and Methods:**

In this experimental study,the cultured spheroids with 100µm diameter were treated by 1 µM IUdR, 43°C hyperthermia for an hour and 2 Gy gamma rays, respectively. The DNA damages induced in cells were compared using alkaline comet assay method, and dosimetry was then performed by TLD-100. Comet scores were calculated as mean ± standard error of mean (SEM) using one-way ANOVA.

**Results:**

Comparison of DNA damages induced by IUdR and hyperthermia + gamma treatment showed 2.67- and 1.92-fold enhancement, respectively, as compared to the damages induced by radiation alone or radiation combined IUdR. Dosimetry results showed the accurate dose delivered to cells.

**Conclusion:**

Analysis of the comet tail moments of spheroids showed that the radiation treatments combined with hyperthermia and IUdR caused significant radiosensitization when compared to related results of irradiation alone or of irradiation with IUdR. These results suggest a potential clinical advantage of combining radiation with hyperthermia and indicate effectiveness of hyperthermia treatment in inducing cytotoxicity of tumor cells.

## Introduction

Glioblastoma multiforme (GBM) is the most aggressive malignant brain tumor in adults with a poor prognosis. First selection for treatment is surgery and radiation therapy and/or chemotherapy. Patients suffering from a GBM have a median survival of less than a year after diagnosis (1).

According to the small number of patients cured using standard therapies, it was found that these tumors are basically resistant to common cancer treatments. Reports have shown as high as 95% for recurrent malignant gliomas after local exci-sion and aggressive adjuvant treatment (2).

To improve the response of patients to radiation therapy, a number of strategies such as the use of radiosensitizing drugs were applied. A halogenated pyrimidine analog iododeoxyuridine (IUdR) which competes with thymidine for incorporation into DNA is identified to sensitize cultured cells to ionizing radiation (IR). Radiosensitizers are usual adjuvant treatments applied before, during and after radiation treatment. These substances increase the ability of ionizing radiation in killing cancer cells (3). In glioblastoma, tumor cells are intrinsically radio-resistant due to poor expression of p53 and through the down-regulation of p21 (4).

Hyperthermia or thermotherapy (thermaltherapy) known as an adjuvant method in cancer treatment is always used with other methods such as chemotherapy or radiotherapy. In this way, the target tissue temperature will increase up to 113˚F (45˚C) in various ways including using RF waves, microwave, etc.

Previous research has shown that heat can shrink or kill tumor cells by damaging proteins and structures within cells (5), or by making cancer cells more sensitive to the radiation or anticancer drugs, whereas normal cells are protected (6, 7). In other words, hyperthermia exclusively targets tumor cells; therefore, the combination of hyperthermia and radiation therapy or chemotherapy has often shown encouraging results, especially in recurrent cancers (5). However, the molecular mechanisms involved in heat-induced cellular responses are still unknown. It can cause cellular damage such single-strand breaks (SSBs) and double strand breaks (DSBs) (8) by inducing structural alterations and strand breaks in chromatin DNA. The DNA DSBs formation has been considered to be just a component or part of the pathway of events leading to heat-induced cell killing. Many investigators have reported that cellular DSBs are detected in heat-treated cells using alkaline elution methods (9), alkaline unwinding assay (10), and pulsed-field gel electrophoresis methods (11).

One of these methods is single cell gel electrophoresis (SCGE), also known as comet assay, which was developed by N.P. Singh. This assay is used to detect the strand breaks of DNA molecule under highly alkaline condition and in an electric field. Broken ends of the negatively charged DNA molecule travel towards the anode under these conditions. These migrations in the electric field stretch out the ends of damaged molecules and create a tail-end, called tail moment, which its length depends on the amount of damage (12).

To assess the effect of hyperthermia alone and in combination with radiation on human glioblastoma cells, we carried out comet assay on cultured cell line U-87MG after multimodality treatment. Ionizing radiation was then administered immediately (less than one hour) to flasks of cells in logarithmic growth at 200 cGy which were also treated by IUdR as well as hyperthermia.

The multicellular spheroid is a transition model of *in vitro*-*in vivo* which is important for *in vivo* solid tumors (13). There are some physiological differences between cell growth in two dimensional contacts (monolayer) and three-dimensional contact (multicellular tumor spheroids) (14, 15). Two researches conducted on the growth of human glioma cells using these two systems have showed different degrees of sensitivity to IUdR and hyperthermia (15, 16), while other studies have showed higher radioresistance of cells in spheroids as compared with monolayer cultures (17-20). In this study, we aimed to evaluate the radiosensitivity of glioblastoma cell line U87MG multicellular spheroids with 100 μm diameter using IUdR in combination with hyperthermia treatment before irradiation by gamma rays. Also for determining the accuracy of radiation absorbed dose in cellular under experimental condition, the TLD-100 dosimeters were applied. So absorbed dose in cellular was measured and compared to given calculated dose.

## Materials and Methods

### Cell line

Human glioblastoma cell line U87MG was provided by Pastor Institute, Tehran, Iran. Cells were maintained in minimum essential media (MEM, Gibco/Invitrogen, USA) supplemented with 500U/ ml of penicillin/ 200 mg/l of streptomycin (Sigma- Aldrich, USA) and 10% fetal bovine serum (FBS, Gibco/Invitrogen, USA).

### Monolayer culture

Monolayer cell culture was performed at a density of 10^4^ cells/cm^2^ in T-25 tissue culture flasks (NUNC, USA) and maintained in MEM supplemented with 10% FBS. Cultures were incubated at 37˚C in a humidified atmosphere of 5% CO_2_. Cells were harvested by trypsinization method using 1mM ethylenediaminetetraacetic acid (EDTA)/0.25% trypsin (PAA, Austria) in phosphate buffer saline (PBS, PAA, Austria) and were sub-cultured weekly.

### Spheroid Culture

Using the liquid-overlay technique (20), spheroids were cultured. Subsequently 105 U87MG cells were added into 100 mm dishes coated with a thin layer of 1% agar [2% agar+ modified eagle medium (MEM) 2X] with 10ml of MEM supplemented with 10% FBS. The plates were incubated at 37˚C in a humidified atmosphere of 5% CO_2_. In half of the cultures, medium was replaced with fresh medium twice a week.

### Spheroid Growth curve

Three days after the primary culture, spheroids were moved into multi-well plates (24 wells/plate) (NUNC, USA) coated with a thin layer of 1% agar. Then 1 ml of MEM supplemented with 10% FBS was added to each well, whereas one spheroid was located in one well. The multi-well was incubated at 37˚C in a humidified atmosphere of 5% CO_2_. Two perpendicular diameters of spheroids were measured for a period of 35 days, while the volume was calculated using the following equation:

$$\mathrm{V (Volume)}=a\times b^{2}\times \frac{\pi }{6}$$ 1

That (a) and (b) are lesser and greater diameters, respectively. Then the volume growth curve was calculated according to the duration. In the logarithmic phase of curve, spheroids follow the equation:

$$V=\mathrm{V0}\times e^{\mathrm{kt}}$$ 2

Where (V0) is primary spheroids volume, (V) equals to the spheroids volume after the duration (t) and k shows the gradient of the logarithmic phase of the curve. The volume doubling time (VDT) of spheroids was achieved from this equation:

$$\mathrm{VDT/}=\frac{\mathrm{Ln2}}{K}$$ 3

### Cell preparation

The 100 μm diameter formed spheroids were divided into 8 groups and coded as following order: A. Control, B. IUdR, C. Hyp, D. IUdR + Hyp, E. Gamma, F. IUdR + Gamma, G. Hyp + Gamma, and H. IUdR + Hyp + Gamma.

### Drug treatment

Four groups coded as B, D, F and H were treated with IUdR with concentration of 1 μM in MEM containing 10% FBS, 63 hour before being exposed to hyperthermia and ionizing radiation. After the treatment time, the medium containing drugs was removed and the cultures were then washed with PBS.

### Hyperthermia and Irradiation

Exponentially growing spheroids coded as C, D, G and H were immediately immersed in a water bath (Memmert, Germany) and maintained at a constant temperature (43℃) for an hour.

Gamma-ray irradiation was administered with a ^60^Co source (Theratron 760, Theratronics, Inc., Ottawa, Canada) for E, F, G and H groups at a dose rate of 86.58 cGy/minute for 2 Gy. For radiation treatment, 4 cell culture flasks were put under collimator of equipment at 80cm distance of the head, simultaneously, while the field size and the period of irradiation were 20×20 cm^2^ and 2.31 minutes, respectively.

### Comet assay

DNA damages include DSBs and SSBs in U87MG cell populations induced either by IUdR alone (1 μM for 63 hours) or in combination with hyperthermia and radiation, determined by comet assay. This assay was performed according to the Singh et al. (12) protocol. We prepared two slides for each sample containing 10^4^ cells. Comet tail moment was determined by measuring the fluorescence intensity using the comet score software. This software provides a measure, referred to tail moment, which has been described as the product of the tail length and the intensity of DNA in the tail of the comet (21).

### TL Dosimetry

The TLD-100 (LiF: Mg, Ti) (Harshaw, Thermo scientific, USA) detectors were simultaneously irradiated with the same conditions used for cells irradiation. The characteristics of these detectors were as follows: dimension of 1.3×1.3×0.9 mm, density of 2.64 g.cm^-3^, and effective atomic number of 8.2 (tissue equivalent dosimeter).

The annealing procedure used for TL dosimeters consists of heating the thermoluminescent dosimeters (TLD) chips at 400˚C for an hour using an annealing oven (Atash-1200, Exciton, Iran) and subsequently heating for 24 hours at 80˚C. Although dosimeters and irradiation condition were similar, due to different sensitivity, the readings were different. To correct this difference, we calculated the Element Correction Coefficient (ECC) which is the ratio of the TL efficiency of a specific dosimeter to the average TL efficiency of the all dosimeters:

$${\mathrm{ECC}}_{i}=\frac{\mathrm{<TLR>}}{{\mathrm{TLR}}_{i}}$$ 4

That ECCi is the ECC of ith dosimeter, <TLR> is the mean TL response (TLR) of the all dosimeters, and TLRi is the TLR of ith dosimeter.

During irradiation, the TLDs were protected by the polystyrene covers while placing in the medium, and they were also placed on a 0.5 cm perspex slab to establish the same conditions which cells were undergoing.

After irradiation, read-out procedure was performed using a TLD-reader (Model QS 3500, Harshaw/Bicron, USA). The read-out values were multiplied by calibration factor achieved from calibration curve and converted to absorbed dose. According to the International Commission of Radiation Units and Measurements (ICRU) and previous studies (22), the uncertainty in the dose determination by TLD-100 should be within ± 5% or lower, in the clinical radiotherapy.

### Statistical analysis

The results of the comet score (tail moments) were calculated as mean ± standard error of mean (SEM). One-way analysis of variance (ANOVA) method was applied to analysis the data. A value of p≤0.05 was considered to be significant.

## Results

**Fig 1:**
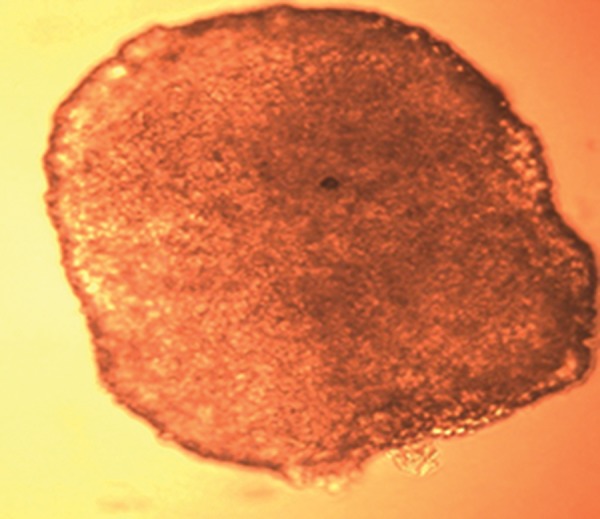
A phase contrast micrograph of U87MG spheroid with 100 μm diameter which was recorded 11 days after initial measurement of diameters (×20).

The U87MG cells were formed as spheroids in liquid- overlay cultures. Figure 1 shows a phase contrast micrograph of the spheroid in 100 μm diameter. VDT for spheroids with 100 μm diameter obtained from growth curve was approximately 63 ± 0.75 hours which were applied as drug-treatment time.

For evaluation of the radiosensitization effects of IUdR and hyperthermia, spheroids were treated with IUdR for 1VDT and subsequently by hyperthermia for an hour. After combined treatment using IUdR, hyperthermia and ionization radiation, comet assay were performed for assessment of DNA damages and values of tail moments shown in table 1.

**Table 1:** Mean tail moment of cells was measured using comet score software and SE values of samples


Group	Mean tall moment	Mean tall moment

**Control**	0.3765	0.0664
**IUdR**	0.7525	0.0356
**Hyp**	1.017	0.0425
**IUdR + Hyp**	1.6727	0.105
**Gamma ^60^Co**	2.2909	0.0881
**IUdR + Gamma ^60^Co**	3.1856	0.1398
**Hyp + Gamma ^60^Co**	3.842	0.1237
**IUdR + Hyp + Gamma ^60^Co**	6.1202	0.1384


IUdR; Iododeoxyuridine and Hyp; hyperthermia.

### Representative samples include following slides

A. Control, B. IUdR, C. Hyp, D. IUdR + Hyp, E. Gamma, F. IUdR + Gamma, G. Hyp + Gamma, and H. IUd R+ Hyp + Gamma.

Microphotographs which were shown in figure 3 display an ascending amount in tail moments and number of damages from A to H.

According to photos, the longest tails of comets were observed in the combined group of IUdr + hyperthermia + radiation, while the shortest tails belonged to control group.

As shown in the figure 4 hyperthermia alone and IUdR alone are not effective enough in changing IR-related cytotoxicity. In contrast IUdR and hyperthermia treatment before IR significantly (p<0.05) increased the tail moments of damaged cells exposed to gamma rays as compared with the control group. But the most DNA damages were observed in the group which was irradiated after treatment with IUdR and hyperthermia.

In order to determine the absorbed dose by cells, TLDs were irradiated simultaneous with cells. Then each TLD was read, its TL reading was multiplied by its ECC, and the background radiation was subtracted. The mean of the values were obtained, the standard deviations (SD) were calculated, and calibration curve was plotted. The calibration factor obtained from curve was multiplied by TL readings in μC unit to convert to absorbed dose in cGy unit.

**Fig 2:**
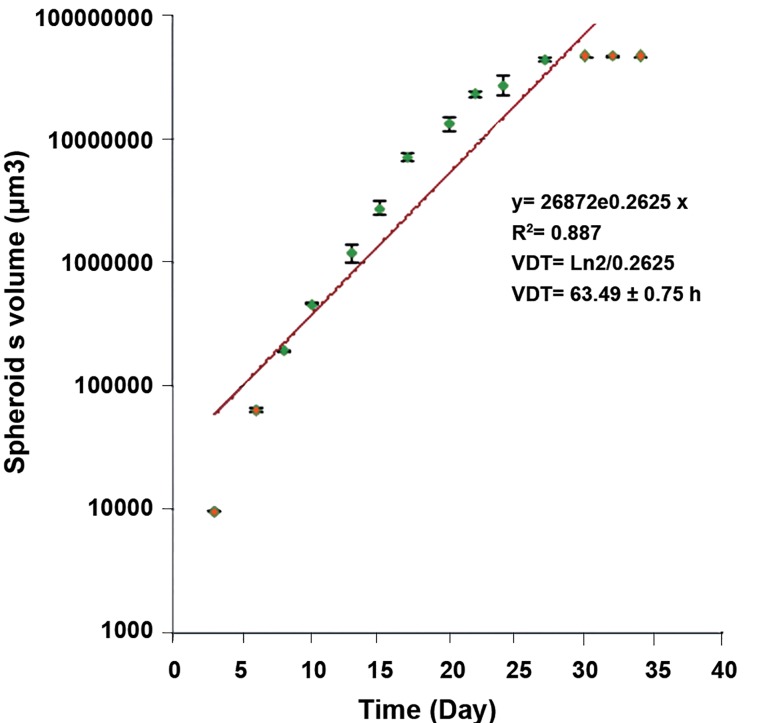
VDT curve of U87MG cell line in the spheroid cultures. The days 6 to 27 showing the log phase of curve were used to measure the VDT (63.49 ± 0.75 hour). The points indicate Mean ± SEM of 3 separate experiment.

**Fig 3:**
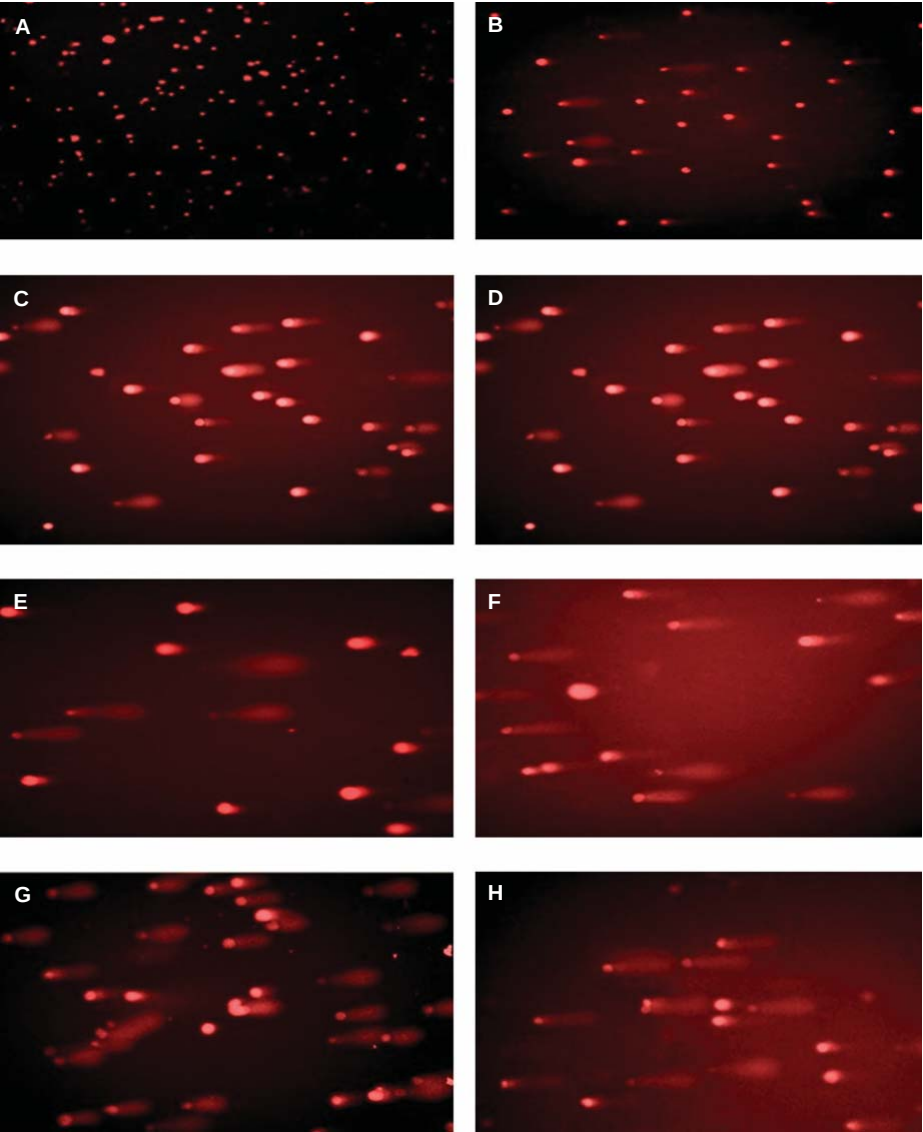
Microphotography of comet assay of U87MG cells of 100μm spheroids.

**Fig 4:**
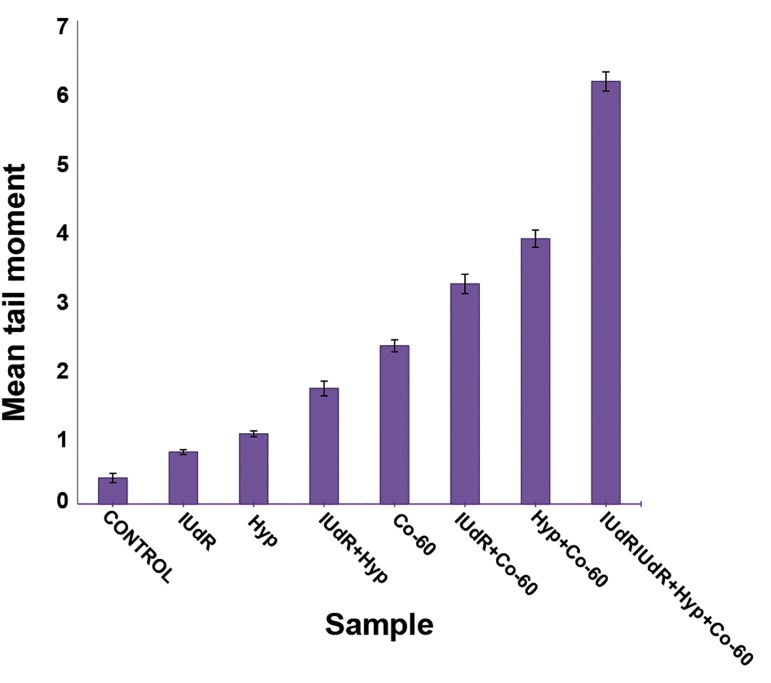
Comparative chart of comet tail moment in U87MG cell line of 100 μm spheroids for various combinations of treatment with 1μM IUdR, hyperthermia (at 43˚C for an hour) and irradiated by gamma rays of cobalt-60. Mean ± SEM of the three separate experiments are plotted. Lines represent fitted polynomial on the data.

## Discussion

Hyperthermia has been used in the therapy of malignant brain tumors for several years. According to the previous studies, hyperthermia affects cell membranes in such a way that normal cells are not damaged, but makes tumor cells more sensitive to radiation. In a study by Pu et al., they have indicated that apoptotic cell death is one of the mechanisms of hyperthermic therapy for malignant glioma (23). Several other studies have demonstrated the survival benefit of hyperthermia in glioma. Some studies applying hyperthermia alone (24, 25) and some others applying adjuvant hyperthermia, given before and after brachytherapy, conventional external radiotherapy (26) or chemotherapy (27), have shown a significant improvement in survival rate of patients with focal glioblastoma. Van Bree et al. Reported supposition about the role of hyperthermia in the treatment of tumors based on the combined use of hyperthermia and radiation therapy, suggesting that hyperthermia probably exacerbates the antitumor effects of radiation as compared to radiation therapy alone. This means that hyperthermia can be effective as an adjunct method in cancer therapy because of ability to provide the lower doses of radiation, especially for highly radioresistant tumors (28). These encouraging results suggest the need for further evaluation of these combined modalities *in vitro* to determine normal tissue toxicity and therapeutic effectiveness.

In the present study, we used IUdR, known as a most potent radiosensitizer due to its reduced toxicity (28), which increased IR-induced DNA damages (29). Spheroids were also used as a tumor model system to assess the influence of hyperthermia and sensitizers on *in vivo* radiation response of cells.

## Conclusion

Comet assay (Fig 3) was applied to evaluate the cytotoxicity effects of the radiation sensitizer in multicell spheroids potentiated by hyperthermia. Enhanced comet tail factor response in cells exposed for one hour at 43˚C indicated DNA damage.

The effect of hyperthermia and IUdR was 2.2- fold as compared to IUdR group, whereas it was demonstrated that hyperthermia and IUdR pretreatment sensitized cells to IR by 1.7-fold and 1.4-fold, respectively, in comparison with radiation alone. However, the most significant cytotoxicity occurred in cells when hyperthermia and IUdR were used together before irradiation. The effectiveness of this combination was 2.7 times greater than when cells were irradiated without treatment.

These results show that a combination of hyperthermia and low concentrations of radiosensitizer can interact positively to induce a high degree of damages in multicell spheroids. This interaction may have several potential therapeutic advantages. The possibility of clinical implication of using three different modalities simultaneity depends on that all equipment used are aggregated in one place.

According to the dosimetry results, the uncertainties of TL dosimeters were about 2.9% (1 SD) which can be due to several factors such as temperature or moisture, low quality of the chips, blowing the chips while transferring to flasks or TLD-reader, or interval between radiation and reading. This uncertainty was found to be high but not essentially greater than its limitation (22). Several studies applied gamma sources and TLD-100 obtained the value of 1.2 and 1.7%, respectively (30, 31).

Therefore, the accuracy of TLD-100 used in this experiment shows that the absorbed dose by cells is in accordance with irradiated dose and considered a desirable result for clinical works.

## References

[1] Sigmond J, Honeywell RJ, Postma TJ, Dirven CM, de Lange SM, van der Born K (2009). Gemcitabine uptake in glioblastoma multiforme: potential as a radiosensitizer. Ann Oncol.

[2] Bashir R, Hochberg F, Oot R (1988). Regrowth patterns of recurrent glioblastoma multiforme related to planning of interstitial brachytherapy radiation fields. Neurosurgery.

[3] Phillips TL, Levin VA, Ahn Dk, Gutin PH, Davis RL, Wilson CB (1991). Evaluation of bromodeoxyuridine in glioblastoma multiforme: a Northern California Cancer Center phase II study. Int J Radiat Oncol Biol Phys.

[4] Furnari FB, Fenton T, Bachoo RM, Mukasa A, Stommel JM, Stegh A (2007). Malignant astrocytic glioma: genetics, biology, and paths to treatment. Genes Dev.

[5] Hildebrandt B, Wust P, Ahlers O, Dieing A, Sreenivasa G, Kerner T (2002). The cellular and molecular basis of hyperthermia. Crit Rev Oncol Hematol.

[6] Van der Zee J (2002). Heating the patient: a promising approach?. Ann Oncol.

[7] Wust P, Hildebrandt B, Sreenivasa G, Rau B, Gellermann J, Riess H (2002). Hyperthermia in combined treatment of cancer. Lancet Oncol.

[8] Roti Roti JL (1982). Heat-induced cell death and radiosensitization: molecular mechanisms. Natl Cancer Inst Monogr.

[9] Warters RL, Henle KJ (1982). DNA degradation in chinese hamster ovary cells after exposure to hyperthermia. Cancer Res.

[10] Dikomey E (1982). Effect of hyperthermia at 42°C and 45°C on repair of radiation-induced DNA strand breaks in CHO cells. Int J Radiat Biol.

[11] Anai H, Maehara Y, Sugimachi K (1988). In situ nick translation method reveals DNA strand scission in HeLa cells following heat treatment. Cancer Lett.

[12] Singh NP, McCoy MT, Tice RR, Schneider EL (1988). A simple technique for quantitation of low levels of DNA damage in individual cells. Exp Cell Res.

[13] Durand RE, Olive PL (1992). Tumour cell kinetics and heterogeneity: insights from multicell spheroids. BJR Suppl.

[14] Wigle JC, Sutherland RM (1985). Increased thermoresistance developed during growth of small multicellular spheroids. J Cell Physiol.

[15] Dobrucki J, Bleehen NM (1985). Cell-cell contact affects cellular sensitivity to hyperthermia. Br J Cancer.

[16] Neshasteh-Riz A, Mairs RJ, Angerson WJ, Stanton PD, Reeves JR, Rampling R (1998). Differential cytotoxicity of (123I) IUdR, (125I) IUdR and (131I) IUdR to human glioma cells in monolayer or spheroid culture: effect of proliferative heterogeneity and radiation cross-fire. Br J Cancer.

[17] Kerbel RS, Rak J, Kobayashi H, Man MS, St Croix B, Graham CH (1994). Multicellular resistance: a new paradigm to explain aspects of acquired drug resistance of solid tumors. ColdSpring Harb Symp Quant Biol.

[18] Olive PL, Durand RE (1994). Drug and radiation resistance in spheroids: cell contact and kinetics. Cancer Metastasis Rev.

[19] Desoize B, Gimonet D, Jardiller JC (1998). Cell culture as spheroids: an approach to multicellular resistance. Anticancer Res.

[20] Desoize B, Jardillier JC (2000). Multicellular resistance: a paradigm for clinical resistance. Crit Rev Oncol Hematol.

[21] Fairbairn DW, O'Neill KL (1996). The neutral comet assay is sufficient to identify an apoptotic 'window' by visual inspection. Apoptosis.

[22] Kirby TH, Hanson WF, Johnston DA (1992). Uncertainty analysis of absorbed dose calculations from thermoluminescence dosimeters. Med Phys.

[23] Pu P, Zhang Z, Jiang H (2000). Apoptosis induced by hyperthermia in human glioblastoma cell line and murine glioblastoma. Chin J Cancer Res.

[24] Sahinbas H, Gronemeyer DHW, Boecher E, Szasz A (2007). Retrospective clinical study of adjuvant electro-hyperthermia treatment for advanced brain gliomas. Germany journal of Oncology.

[25] Silva AC, Oliveira TR, Mamani JB, Malheiros SM, Malavolta L, Pavon LF (2011). Application of hyperthermia induced by superparamagnetic iron oxide nanoparticles in glioma treatment. Int J Nanomedicine.

[26] Sneed PK, Stauffer PR, McDermott MW, Diederich CJ, Lamborn KR, Prados MD (1998). Survival benefit of hyperthermia in a prospective randomized trial of brachytherapy boost +/- hyperthermia for glioblastoma multiforme. Int J Radiat Oncol Biol Phys.

[27] Baronzio GF, Mainini C, Fiorentini G, Guais A, Schwartz L (2010). Astrocytomas glioblastomas hyperthermia metabolic inhibitors.Some considerations. Oncothermia Journal.

[28] Van Bree C, Franken NA, Bakker PJ, Klomp-Tukker LJ, Barendsen GW, Kipp JB (1997). Hyperthermia and incorporation of halogenated pyrimidines: radiosensitization in cultured rodent and human tumor cells. Int J Radiat Oncol Biol Phys.

[29] Watanabe R, Nikjoo H (2002). Modeling the effect of incorporated halogenated pyrimidine on radiation-induced DNA strand breaks. Int J Radiat Biol.

[30] Izewska J, Hultquist M, Bera P (2008). Analysis of the uncertainties in the IAEA/WHO TLD postal dose audit system. Radiat Meas.

[31] Sawa A (2010). Personnel TLD monitors their calibration and response.Presented for M.Sc..

